# Study on the Diversity of Fungal and Bacterial Communities in Continuous Cropping Fields of Chinese Chives (*Allium tuberosum*)

**DOI:** 10.1155/2020/3589758

**Published:** 2020-12-17

**Authors:** Yizhu Gu, Yuxin Wang, Pingzhi Wang, Chaonan Wang, Jinhai Ma, Xiaofei Yang, Donghao Ma, Meihuan Li

**Affiliations:** ^1^College of Water Resources & Civil Engineering, China Agricultural University, Haidian, Beijing, China; ^2^Henan Jiuxing Institute of Biotechnology, Pingdingshan, Henan, China

## Abstract

In this study, high-throughput sequencing technology was used to analyse the diversity and composition of fungal and bacterial communities in continuous cropping soil of Chinese chives. The soil nutrient was also measured to explore the rationality of current fertilization management. These results can provide a basis for the prevention and control of the continuous cropping obstacles of Chinese chives and further scientific management. Soil samples from fields continuously cropped with Chinese chives for one year, three years, and five years were collected and analysed. The results showed that the nutrient content of TP, AP, AK and TK increased significantly with increasing continuous cropping years. Short-term continuous cropping soil nutrients have not deteriorated. Alpha-diversity analysis showed that significant differences were not found in the diversity of the fungal and bacterial community among different years. *Ascomycota, Basidiomycot*a and *Mortierellomycota* were the three most dominant fungal phyla. *Proteobacteria, Actinobacteria, Chloroflexi* and *Acidobacteria* were the dominant bacterial phyla. Continuous cropping makes *Fusarium* increase, and the beneficial bacteria *Pseudomonas* decreased significantly. According to the correlation heat map analysis of environmental factors, excessive phosphorus may lead to the increase of Fusarium, potassium may promote the proliferation of beneficial bacteria in the continuous cropping process, and it is necessary to regulate the application of phosphate and potassium fertilizer.

## 1. Introduction

Chinese chives are perennial plants that belong to the vegetable of Liliaceae, and continuous cropping is a traditional planting method for Chinese chives. More recently, an increasing number of studies have speculated that continuous cropping or consecutive monoculture results in imbalances in the soil microbial community diversity and structure [1–3]. Changes in the soil microbial biomass, diversity, and microflora directly affect soil health, which in turn affects crop growth [4, 5]. Many fruits and vegetables have cropped continuously, such as Cucumis sativus, Capsicum annuum, Lycopersicon esculentum, and Solanum melongena, which cause serious pests and diseases and lower yields. Continuous cropping obstacles are a common problem in the cultivation of crops and traditional Chinese medicinal herb, which is related to the root system, soil nutrients, antagonism, and microbial flora [6]. Long-term continuous cropping will cause soil deterioration, soil fertility decline, and increase the occurrence of diseases and insect pests, affecting the yield and quality of Chinese chives [7]. The mechanism and causes of continuous cropping obstacles are still unclear. Most farmers in the planting areas mainly use pesticides and fertilizers to maintain crop yields, but the results are often unsatisfactory and increase production costs, pollute the environment even bring about the degradation of farmland ecosystem functions, etc. [8].

Wu et al. [9] found that the continuous cropping of *Rehmannia glutinosa* resulted in a reduction in the number of rhizosphere soil bacteria and an increase in soil fungi, and the soil-borne pathogens Fusarium and Rhizoctonia increased continuously in the rhizosphere soil with the continuous cropping years; beneficial Pseudomonas and Bacillus gradually reduced. Microorganisms are key factors in the rhizosphere microecological system and play an important role in the soil material cycle. The interaction between plants and soil is generally regulated by soil microbes [10]. The accumulation of phenolic acid in the rhizosphere of some plants under continuous cropping conditions leads to changes in the soil microbial ecological environment [11, 12]. Continuous cropping can also reduce the resistance of soil microbial communities to the external environment. With the increase in continuous cropping years, soil is transformed from bacterial-dominated to fungal-dominated, and pathogen antagonistic bacteria are reduced [13, 14], which may be the main feature of continuous cropping [15]. At the same time, some studies have shown that the same crops also have specific demand types and absorption ratios of nutrients in the soil. Long-term single planting will lead to unbalanced soil nutrients [16].

The responses of different crops to continuous cropping vary. There is little research on the obstacles of continuous cropping of Chinese chives thus far. Gang et al. [7] found that fungi decreased, *Actinomycetes* increased sharply, and the total amount of microorganisms decreased in continuous cropping of Chinese chive fields. Continuous cropping of Chinese chives can easily cause black root disease, which can harm the stems and leaf sheaths of Chinese chives. Currently, soil-borne diseases of Chinese chives caused by fungi include bulb rot (Kampu-byo), which is a serious disease of Chinese chives caused by *Fusarium oxysporum* and *F. moniliforme* [17]. In addition, the gray mold is also one of the common diseases of Chinese chives, which is caused by the infection of *Botrytis cinerea* and mostly occurs in winter and spring in low temperature and rainy years. The relationship between fungal pathogens and continuous cropping is few to research. Only a portion of microorganisms have been detected in previous studies using culture-dependent or molecular analyses, such as DGGE and q-PCR, thereby precluding a full understanding of soil microbes [18–20]. Recently, the development of high-throughput sequencing has helped to fully elucidate the soil microorganisms and better understand their relationship with plant disease [21]. Researchers can simultaneously sequence a variety of microbial genomes and study the richness and diversity of soil microorganisms to help better study the relationship between microbes and crop growth and provide data support for overcoming continuous cropping obstacles.

There are abundant Chinese chive resources in Pingdingshan City, Henan Province, China. The Institute of Horticulture has been engaged in the research of breeding Chinese chives all year round, and approximately one-fifth of the new Chinese chive varieties in China were bred here. The continuous cultivation of Chinese chives for many years is easy to cause continuous cropping obstacles, but the composition and structure of the soil microbial community of continuously cropped Chinese chives are still unclear. In this study, we selected soil from Chinese chive fields that have been continuously cropped at the Horticulture Institute for a different number of years. By high-throughput sequencing technology, we analysed the changes in soil bacterial and fungal microbial community structures to explore the effects of continuous cropping on the soil microecological environment of Chinese chives from the perspective of microbial community change.

## 2. Materials and Methods

### 2.1. Site Description and Soil Sampling

The sampling site of the experiment was located at Pingdingshan Institute of Horticultural Science, Henan Province, China (latitude, 33.34°N; longitude, 113.03°E). Three Chinese chive fields were selected for this study, in which the soil samples were collected from the fields where Chinese chives (variety: Jiuxing) had been continuously planted for 1, 3, and 5 years (referred to as “YC1,” “YC3,” and “YC5,” respectively). These Chinese chives were arranged in the same ecological area and conducted under similar field conditions and fertilizer management. Before planting Chinese chives, the growers apply sufficient base fertilizer and plow the land. Planting is in early April, with an average daily temperature of 12-20°C, adopting seedling transplanting methods, and the thickness of the cover soil is generally 10-15 cm. During the growth period of Chinese chives, organic fertilizers are mainly used, and nitrogen, phosphorus, and potassium compound fertilizers are supplemented. The commercial characteristics of the Jiuxing series of Chinese chives we studied are outstanding, mainly including low crude fiber content, tolerance to storage and transportation, high resistance to gray mold, phytophthora disease, and strong growth potential. The height of the Chinese chive is more than 40 cm, the sheath length is more than 12 cm, and the sheath thickness is 0.8 cm. The average weight of each Chinese chive is more than 10 g. Chinese chives can be harvested more than 7 times a year, and the total yielding can reach 13000 kg per 667 m^2^.

Corresponding to the field of Chinese chive with specific years of cultivation, 5 cores (0–20 cm in depth and 2.5 cm in diameter) of soil were randomly collected after removing surface coverings, and 5 random cores of soil were thoroughly mixed to make a composite sample. All 9 soil samples (3 fields × 3 samples) were stored into sterile plastic bags, placed on ice, and transported to the laboratory. After passing through a 100-mesh sieve, we divided the samples into two halves. One part of each sample was stored in an ultralow temperature freezer at -80°C for subsequent DNA extraction, and the remaining part was air-dried to determine the soil nutrient content.

### 2.2. Determination of Soil Physicochemical Properties

The soil total nitrogen (TN) contents were measured using an elemental analyser (Vario EL III, Elementar, Hanau, Germany) [22]. Soil total phosphorus (TP) was digested with H_2_SO_4_-HClO_4_, and available phosphorus (AP) was extracted with 0.5 M NaHCO_3_ [23]. Soil total potassium (TK) was digested with HNO_3_-HClO_4_-HF, and available potassium (AK) was measured by flame photometry after NH_4_OAc extraction [24].

### 2.3. DNA Extraction and Sequencing

Microbial communities were analysed according to DNA extraction, PCR amplification, and Illumina MiSeq sequencing procedures.

Total bacterial DNA was extracted from 500 mg of soil using the Fast DNA SPIN Kit for Soil according to the protocol. The bacterial universal V3-V4 region of the 16S rRNA gene was amplified with the primers 338F (5′-ACTCCTACGGGAGGCAGCAG-3′) and 806R (5′-GGACTACHVGGGTWTCTAAT-3′). The fungal universal ITS region was amplified with the primers ITS1-F (5′-CTTGGTCATTTAGAGGAAGTAA-3′) and ITS2 (5′-TGCGTTCTTCATCGATGC-3′) [25].

PCR amplification was performed in a total volume of 20 *μ*l, containing 4 *μ*l of 5x FastPfu Buffer (for the bacterial V3-V4 region) or 2 *μ*l of 10x Buffer (for the fungal ITS region), 2 *μ*l of 2.5 mM dNTPs, 0.8 *μ*l of 5 *μ*M forward primer, 0.8 *μ*l of 5 *μ*M reverse primer, 0.4 *μ*l of Fast Pfu Polymerase (for the bacterial V3-V4 region) or 0.2 *μ*l of rTaq Polymerase (for the fungal ITS region), 0.2 *μ*l of BSA, and 10 ng of template DNA. PCR amplification with a ABI GeneAmp® 9700 PCR system was conducted according to the following conditions: 3 minutes at 95°C; 37 cycles (for the fungal ITS region) or 27 cycles (for the bacterial V3-V4 region) of 30 s at 95°C, 30 s for annealing at 55°C, and 45 s for elongation at 72°C; a final extension at 72°C for 10 min; and finally, 10°C until the reaction was halted by the user.

All PCR amplicons were visualized on 2% agarose gels. The DNA concentration of each PCR product was determined by the QuantiFluor™-ST fluorescent quantitative system (Promega, USA) before sequencing. High-throughput sequencing of amplicons was performed using the Illumina MiSeq platform by Shanghai Majorbio Bio-pharm Technology Co., Ltd.

The high-quality sequences were clustered into operational taxonomic units (OTUs) at a threshold of 97% similarity using the UPARSE pipeline, and chimeric sequences were identified and removed by means of UCHIME. The taxonomy of each ITS gene sequence was analysed by RDP Classifier (http://rdp.cme.msu.edu/) against the ITS database UNITE (release 8.0) using a confidence threshold of 70% [26]. Similarly, the taxonomy of each 16S rRNA gene sequence was analysed by the RDP Classifier algorithm (http://rdp.cme.msu.edu/) against the SILVA (Release 138) 16S rRNA database with a confidence threshold of 70% [27].

### 2.4. Statistical Analysis

Alpha-diversity analyses, including community richness indices (Chao1, ACE), community diversity indices (Shannon, Simpson), and a sequencing depth index (Good's coverage), were calculated with the assistance of Mothur software [28]. Rarefaction curves were generated based on the observed species richness. A Venn diagram was used to display the common and unique OTUs among the three soil groups [29]. The parameters were calculated using one-way analysis of variance (ANOVA) and Wilcoxon rank sum test [30]. The R language tool was used to map the community histograms showing the relative abundance of each microorganism at the phylum level, and the R language vegan package was used to draw community heat maps to reflect the similarities and differences of the community compositions of different samples at genus levels by colour change. The taxonomic dissimilarity analysis between samples was performed based on the PCoA method and hierarchical clustering tree with Bray-Curtis distances (beta diversity) [31]. Pearson's correlation analysis was performed between the top 15 genera of fungi and bacteria (based on the rank of relative abundance) environmental variables, and corresponding heat maps were produced in R project with the “heat map” package. The one-way ANOVA was used to perform the difference figure and table between the top 15 genera of fungi and bacteria with significant difference (based on the rank of relative abundance), as well as the difference figure of phylum levels of bacteria and fungi in the three groups (abundance > 1%). The sample data of each group (YC1, YC2, and YC3) is the mean of three replicates of each group.

## 3. Results

### 3.1. Soil Physicochemical Properties

The soil nutrient content in the soil of Chinese chive fields under different continuous cropping years is shown in Table 1. Table 1 shows that the nutrient content of TP, AP, AK, and TK increased significantly with increasing continuous cropping years, especially AP. In addition, TP increased significantly in the fifth year of continuous cropping, and AK increased most significantly in the third year of continuous cropping. All the nutrient content showed significant differences between different continuous cropping years' field.

### 3.2. Bacterial and Fungal Community Diversity

From all 9 soil fungal samples, a total of 610640 high-quality reads were obtained, ranging from 48225 to 74803 reads per sample. In total, 1377 OTUs were clustered, which were grouped into 362-533 OTUs for each sample. In contrast, 527218 high-quality 16S reads were obtained from the bacterial samples, ranging from 45290 to 63513 reads per sample. A total of 2532 OTUs were clustered after random sampling, ranging from 1836 to 2015 OTUs per sample, at a 97% sequence similarity cut-off.

**Figure 1:**
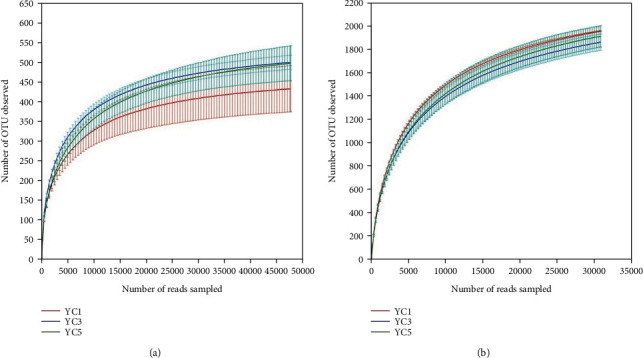
(a) Fungal and (b) bacterial rarefaction curves at 97% similarity levels of the Chinese chives' different continuous cropping soil. The error bars above the scatter points represent the standard deviation of three replicates.

The rarefaction curve can be used to compare the richness, uniformity, or diversity of species in samples with different sequencing data and can also be used to indicate whether the amount of sequencing data in the sample is reasonable. The rarefaction curves of bacteria and fungi were basically flattened with the increase in sequencing data, indicating that the sequencing depth was sufficient, and the rarefaction curves could reflect the bacterial (or fungal) community of the samples from different continuous cropping years (Figure 1). The upper limits of OTUs from YC3 and YC5 were larger than those from YC1 (Figure 1(a)), and the bacterial rarefaction curves of YC3 and YC5 were lower than those of YC1 to some extent (Figure 1(b)), which showed a downward trend in the bacterial community and an increasing trend in the fungi community diversity after one year of continuous cropping. However, the curve was not sufficient to show differences that continuous cropping increased the fungal diversity and decreased bacterial diversity.

The Shannon index and the Simpson index can be used to estimate microbial diversity. The larger the Shannon index or the smaller the Simpson index, the higher the community diversity is. The Sobs, ACE, and Chao indices are used to estimate the number of OTUs and reflect sample richness; the larger values of which indicate a high degree of community richness. The coverage index refers to the coverage of each sample, which reflects whether the sequencing results represent the real situation of microorganisms in the sample.

In this study, Good's coverage index of bacteria and fungi was close to 100% in the different groups, which indicated that the sequencing results at this level can reflect the true situation of the microorganisms in the sample (Table 2). In addition, Table 2 shows no significant difference in *α*-diversity index between the different years' samples, which shows that the diversity of bacteria and fungi did not change significantly in short-term continuous cropping, even if we can see an insignificant change trend from the rarefaction curve (Figure 1).

### 3.3. Bacterial and Fungal Community Composition

**Figure 2:**
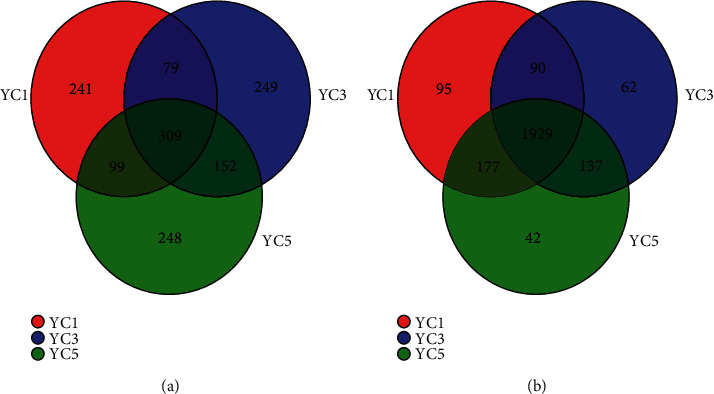
Venn diagram of (a) fungal and (b) bacterial OTUs for the three collected soil samples under continuous cropping of Chinese chives.

A Venn diagram can show the shared and unique microbial taxa between different samples directly. We obtained the number of OTUs in each sample; a total of 1377 fungal OTUs and 2532 bacterial OTUs at 97% similarity were obtained, and the YC1, YC3, and YC5 groups obtained 728, 789, and 808 fungal OTUs, respectively. The number of shared OTUs in the three different groups was 309, accounting for 22.5% of the total number of fungal OTUs (Figure 2(a)). Similarly, the YC1, YC3, and YC5 groups contained 2291, 2218, and 2285 bacterial OTUs, respectively. The total number of shared OTUs in the three different groups was 1929, accounting for 76.2% of the total number of bacterial OTUs (Figure 2(b)), which indicated that the bacterial groups in the three different continuous cropping years were more consistent, and the effect of continuous cropping on the fungal community was greater than that of bacteria.

**Figure 3:**
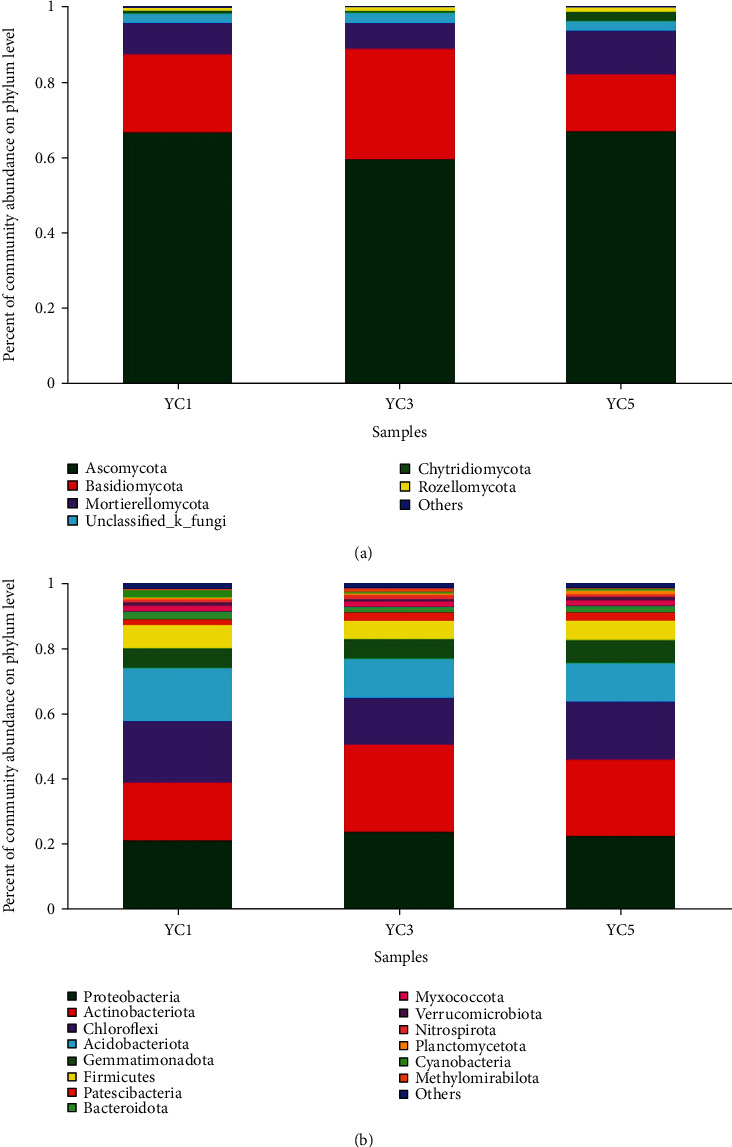
Composition of the different (a) fungal and (b) bacterial communities at the phylum level in different continuously cropped soil samples.

All effective sequences were classified at the phylum level by the Mothur software, and the fungal OTUs were assigned to 13 phyla, 45 classes, 99 orders, 200 families, 375genera, or 542 species. Similarly, the bacterial OTUs were assigned to 37 phyla, 108 classes, 235 orders, 351 families, 590 genera, or 1117 species. Sequences that could not be classified were assigned as no rank, and sequences with abundances less than 1% in all samples were classified as others. There were 6 phyla with relative abundances greater than 1% detected in the fungal samples. The overall fungal composition of the different samples was similar. *Ascomycota, Basidiomycota* and *Mortierellomycota* were the three most dominant phyla. Among them, *Ascomycota* was the most abundant phylum (Figure 3(a)), accounting for approximately 59.34% to 66.78% of the reads across all groups, whereas the relative abundance of *Basidiomycota* and *Mortierellomycota* was 15.1-29.32% and 6.79-11.57% in all groups, respectively. The total abundance of the three phyla in each group could reach more than 90%.

At the phylum level, 14 bacterial phyla (relative abundance > 1%) were detected in the Chinese chive soil samples, including *Proteobacteria, Actinobacteriota, Chloroflexi, Acidobacteriota, Gemmatimonadota, Firmicutes, Patescibacteria, Bacteroidota, Myxococcota, Verrucomicrobiota, Nitrospirota, Planctomycetota, Cyanobacteria* and *Methylomirabilota* (Figure 3(b)). Among them, *Proteobacteria, Actinobacteriota, Chloroflexi* and *Acidobacteriota* were the main bacterial phylum, and each of them the abundance was more than 10%. The abundances of *Proteobacteria* (the most abundant) in YC1, YC3, and YC5 were 21.48%, 24.09%, and 22.86%, respectively. The relative abundances of *Actinobacteriota* in YC1, YC3, and YC5 were 17.69%, 26.57%, and 23.29%, respectively. Similarly, the relative abundances of *Chloroflexi* in YC1, YC3, and YC5 were 18.64%, 14.26%, and 17.60%, respectively, and the relative abundances of *Acidobacteriota* in YC1, YC3, and YC5 were 16.24%, 11.91%, and 11.76%, respectively. Significant difference analysis at the phylum level is shown in Figure S1; only *Basidiomycota* has a significant difference in fungal community, and *Actinobacteriota* has a significant difference in bacterial community (abundance > 1%) (*P* < 0.05).

### 3.4. Comparative Analysis of Sample Community

**Figure 4:**
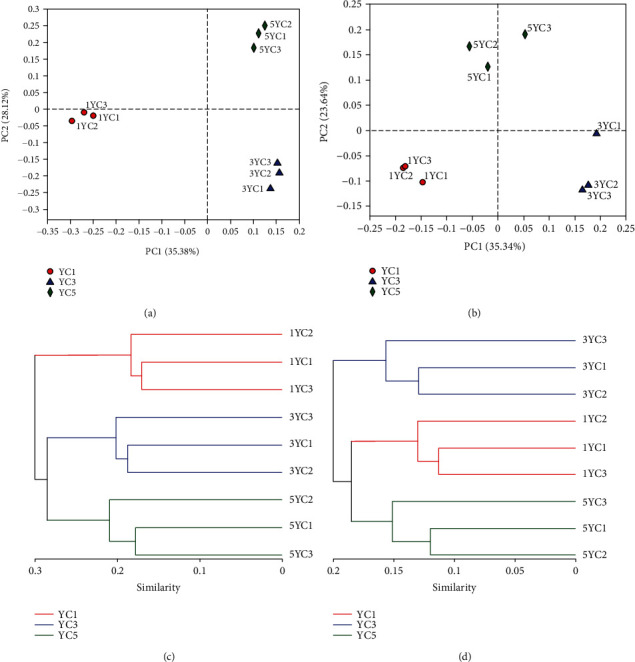
Sample composition structure similarity and difference at the OTU level. (a) Fungal principal coordinate analysis plot based on the Bray-Curtis method; (b) bacterial principal coordinate analysis plot based on the Bray-Curtis method; (c) fungal clustering analysis plots based on the Bray-Curtis method; (d) bacterial clustering analysis plots based on the Bray-Curtis method. The three colours of red, blue, and green represent the three continuous cropping years (YC1, YC3, and YC5, respectively).

PCoA plots were constructed based on the selected distance matrix, and the potential principal components that affect the composition difference of the sample community were identified through dimensionality reduction. PCoA plots do not change the mutual positional relationship between sample points but only change the coordinate system. In this study, PCoA plots using the Bray-Curtis method on OTU abundance were mapped to further analyse the differences in the microbiota of soil from different continuous cropping years (Figures 4(a) and 4(b)). PCoA analysis showed that the variation in the fungal community composition was affected by 8 principal coordinate components. However, the first two main coordinate components had the greatest impact, which could explain 35.38% and 28.12% of the variance, and the cumulative explanatory variation was 63.5%. The bacterial community was also controlled by 8 principal coordinate components. The first two principal components of the bacterial community could explain 35.34% and 23.64% of the variance, and the cumulative explanatory variation was 58.98%. The samples under the same continuous cropping were clustered together and clearly separated from other years. The fungal community of YC1 can be separated from the other two groups by principal component 1, and the fungal community of YC3 and YC5 groups can be separated by the second principal component. Three groups of bacterial communities can be completely separated by principal component 1. Sample cluster analysis uses evolutionary information between individual sample sequences to compare significant microbial community differences in a particular evolutionary lineage using the unweighted pair-group method with arithmetic means (UPGMA) to aggregate the sample class. In this study, we drew a dendrogram based on the Bray-Curtis method. As shown in Figures 4(c) and 4(d), the fungi from the samples of the same continuous cropping year were clustered together, and YC3 and YC5 had higher similarity in the community composition. However, the results of bacteria were different with fungi based on the Bray-Curtis method; the samples of the YC1 group and the YC5 group were clustered into one class first and they had higher homogeneity compared with the sample of YC3.

**Figure 5:**
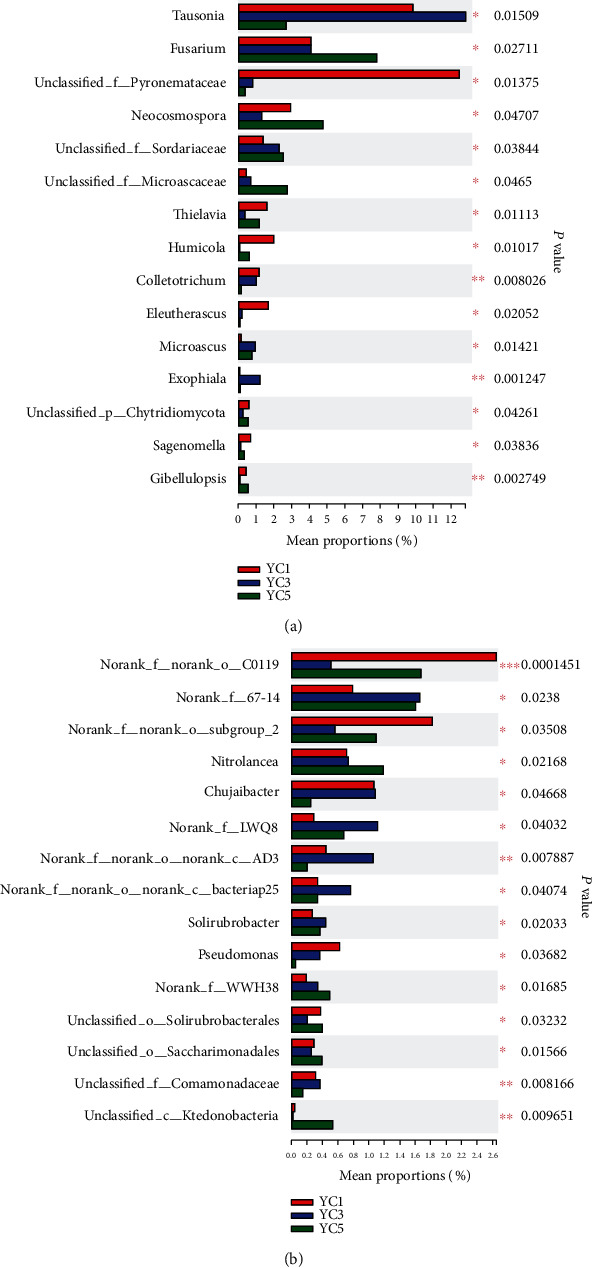
The top 15 (a) fungal and (b) bacterial genera with significant differences. The *y*-axis represents the species name at a certain taxonomic level, p represents phylum, c represents class, o represents order, and f represents family. The *x*-axis represents the average relative abundance in different groupings of species, and the columns with different colours represent different group. Significant differences were identified by means of one-way ANOVA, and *P* values were corrected for multiple comparisons using the false discovery rate (FDR). The *P* value is on the right side; ^∗^0.01 < *P* ≤ 0.05, ^∗∗^0.001 < *P* ≤ 0.01, and ^∗∗∗^*P* ≤ 0.001.

To further analyse the changes in the microbial community structure, we analysed the top 15 fungi and bacteria with significant differences at the genus level (Figures 5(a) and 5(b)). Among the 15 genus level classifications, 4 fungal genera significantly increased with increasing continuous cropping years, including *Fusarium, Neocosmospora, unclassified_f__Sordariaceae* and *Unclassified_f__Microascaceae*, whereas Tausonia, unclassified_f__Pyronemataceae, Thielavia, Humicola, Colletotrichum, and *Eleutherascus* significantly reduced (*abundance < 1%* not discussed). With respect to bacterial communities, *Norank_f__67-14*, *Nitrolancea*, *Norank_f__LWQ8*, *Norank_f__WWH38*, *Solirubrobacte* and *Unclassified_c__Ktedonobacteria* were increased, whereas *Norank_f__norank_o__C0119*, *Norank_f__norank_o__Subgroup_2*, *Chujaibacter* and *Pseudomonas* were decreased. The specific abundance values are shown in Table S1.

### 3.5. Correlation Analysis

**Figure 6:**
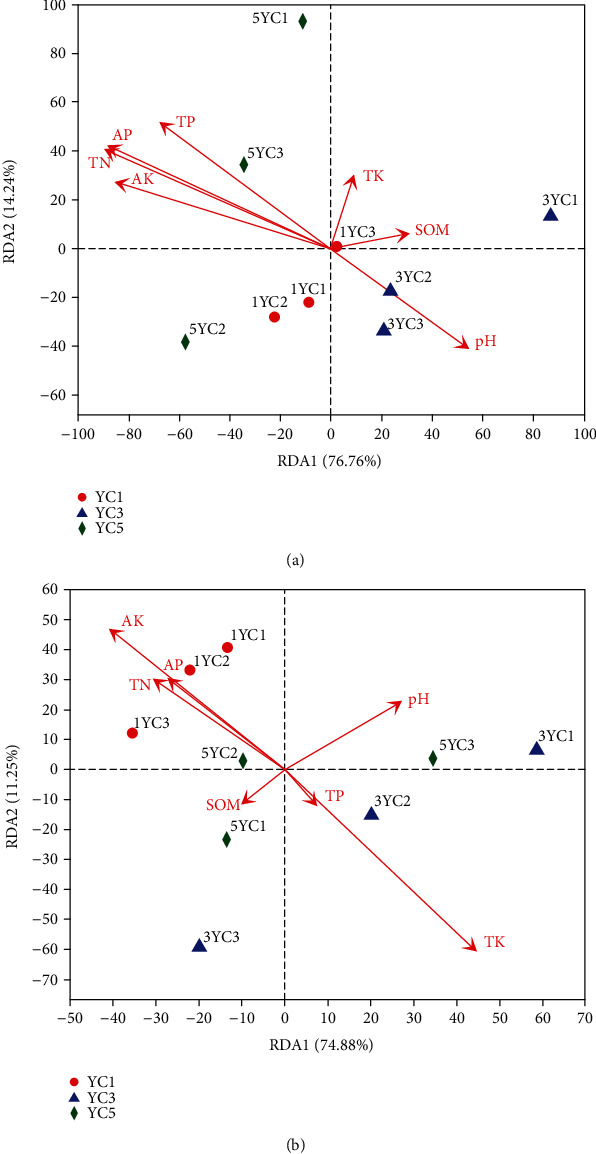
The relationship of microorganism community with the major environmental factors at the phylum level: (a) fungi and (b) bacteria. The environmental factors and different groups are indicated by red arrows and different shapes, respectively.

The relationship of microbial communities with the major environmental factors at the phylum levels was studied in Figure 6. For fungi, the first RDA dimension explained 76.76% of the variation in communities, and the second explained 14.24%. And the environmental factors have little effect on the microbial community in the first year of continuous cropping (YC1). AK, AP, TP, and TN are variables that have a significant influence on the fungal community (*P* < 0.05). For bacteria, the first RDA dimension explained 74.88% of the variation in communities, and the second explained 11.25%. The environmental variable that contributed significantly to the bacteria community-environment relationship was TK (*P* < 0.05).

Pearson correlation analysis shows the effects of soil nutrient content on bacteria and fungi were different (Figure 6). Among the top fifteen genera of fungi abundance, six genera were affected by at least one environmental factor (Figure 6(a)). *Fusarium* and *Acaulium* were significantly positively correlated with TP, and *Fusarium* was also positively correlated with AP. *Neocosmospora* was significantly positively correlated with TN and AP. The *Unclassified_f__Pyronemataceae* was significantly negatively correlated with TK, whereas *Trichoderma* was positively correlated with TK. At the same time, *Tausonia* was significantly negatively correlated with TP, AP, and TN. We observed that AK had no effect on fungal community changes.

Among the top fifteen genera of bacteria abundance (Figure 6(b)), five bacterial genera were associated with environmental factors including *Norank_f__norank_o__Gaiellales*, *Unclassified_f__Xanthobacteraceae*, *Norank_f__norank_o__C0119*, *Norank_f__norank_o__Acidobacteriales*, and *Norank_f__67-14*. The *Norank_f__norank_o__Gaiellales* was significantly correlated with TK, whereas *Norank_f__norank_o__Acidobacteriales* was negatively correlated with TK. *Unclassified_f__Xanthobacteraceae* was correlated with AK. Obviously, the TP and AP had no obvious effect on bacterial community changes.

## 4. Discussion

There are many complex reasons for crop continuous cropping obstacles, such as deterioration of soil physicochemical properties, decrease in soil enzymatic activities, accumulation of autotoxic substances, and build-up of soil-borne pathogens [32–34]. Although there are many reasons for continuous cropping obstacles, they mainly come from the soil. The most fundamental reason is the imbalance of soil microflora and diversity, the decrease in beneficial microorganisms, and the enrichment of pathogenic microorganisms, which lead to various soil-borne diseases of plants [35]. In agricultural ecosystems, soil microbial diversity plays an important role in maintaining ecosystem balance. The soil microbial community is the basis of soil ecological function. It affects the circulation of soil nutrients with regulates and indicates soil function through participating in decomposition and mineralization of soil organic matter [36]. Under the modern single crop cultivation mode, the growth and reproduction rate of pathogenic microorganisms in the crop growth period is much higher than that of traditional rotation mode. With the increase in continuous cropping years, the number of pathogenic microorganisms will eventually exceed the critical value of disease, leading to the occurrence of soil-borne diseases [37]. Soil nutrient content was first measured in our research. All soil variables exhibited significant differences among the three fields by one-way ANOVA (Table 1). N, P, and K nutrient content showed an overall upward trend, which is different from previous studies that continuous cropping can lead to deterioration of soil physical and chemical properties to a certain extent [38, 39]. On the one hand, due to the fact that these study sites were located at geographically proximate areas and possessed similar edaphic textures, agronomic management practices, and fertilization regimes, the increasing of nutrients may be attributed to the sufficient supply of nitrogen, phosphorus, and potassium fertilizers during the five years of continuous cultivation. On the other hand, the increases in soil nutrient contents should be associated with poor uptake of the nutrients by Chinese chives with increasing planting period. The poor uptake of the nutrients by Chinese chives leads to the accumulation of these nutrients in soil. We can conclude that under reasonable management measures, the short-term continuous cropping soil nutrient properties have not been destroyed, but this does not mean that continuous cropping has not affected the soil and crop properties. Continuous cropping may lead to reduced nutrient absorption by crops to a certain extent.

Most research results showed that soil microorganisms were closely related to the continuous cropping, which reflected on increased fungi and decreased bacteria with the increase in continuous cropping years, such as continuous cropping of sorghum reduced the richness of soil bacterial communities and increased fungal diversity [40]. And the richness of soil bacterial communities reduced in the study of potato [41]; there were similar reports about soil fungal diversity increase under continuous cropping of *Casuarina equisetifolia* as well [42]. In our study, there was no significant change in the diversity of bacterial and fungal communities within five years of continuous Chinese chive cropping (Table 2). According to the Venn diagram at the OTU level, the number of shared fungal OTUs in the three different groups was 309, accounting for 22.5% of the total number of fungal OTUs, and the total number of shared bacterial OTUs in the three different groups was 1929, accounting for 76.2% of the total number of bacterial OTUs (Figure 2(b)). Similar studies were also carried out in vanilla-growing soil in which fungal diversity was closely associated with continuous cropping in vanilla-growing soil, but bacterial diversity was not significantly changed [43].

Through studies on the community composition of fungi and bacteria (Figure 3), it was found that the overall microbial compositions of the samples were similar; however, different proportions were observed from some samples at the phylum level. *Ascomycota, Basidiomycota* and *Mortierellomycota* were the dominant fungi in soil, and *Ascomycota* was also the most abundant fungal phylum in the rhizosphere of *Pisum sativum* and banana [44], which is consistent with previous studies [37, 45]. For bacteria, the composition of bacteria at the phylum level was also similar; *Proteobacteria, Actinobacteriota, Chloroflexi* and *Acidobacteriota* were the main bacterial phylum in our research; at the phylum level, *Basidiomycota* and *Actinobacteria* appeared to have significant differences in fungal and bacterial communities, respectively (abundance > 1%) (Figure S1). The abundance of *Actinobacteria* increased, which are known for their production of high levels of secondary metabolites for plant disease suppression and growth promotion [46]. In addition, a positive correlation between *Actinobacteria* levels and plant health was also observed in wheat-cropping region, in Canada's oldest organic-conventional study field [47].

The dominant fungi with higher relative abundance at the genus level were *Tausonia, Chaetomium, Solicoccozyma, Mortierella, Trichoderma, Fusarium, Candida* and *Cryptococcus*, etc. (Figure S2a). Among them, *Fusarium* is the main pathogenic fungus in soil-borne diseases, and *Trichoderma* is an important plant disease-causing fungus that can control the disease by inducing gene expression of the plant immune system. Among the top 15 fungal genera with significant differences (Figure 5), *Fusarium* increased significantly (*P* < 0.05). Many soil-borne diseases were caused by *Fusarium* [48, 49], which contains many pathogenic species; thus, an increase in *Fusarium* abundance is likely to lead to plant disease [50]. *Mortierella* is a beneficial microorganism in soil and can promote plant growth by providing nitrogen nutrition and improving plant disease resistance, which may be related to the sugar metabolism of plants [38]. However, *Mortierella* did not cause a significant change during the five-year continuous cropping period. Among the top 15 fungi genera with significant differences, *Nitrolancea* increased significantly, and *Chujaibacter* and *Pseudomonas* decreased significantly with the continuous cropping years (Figure 5, Table S1). *Pseudomonas* was beneficial microorganisms that can protect banana from *Fusarium wilt* disease [45]. So the beneficial flora has a downward trend in the continuous cropping soil. Most of the top 15 genera of bacterial abundance are not classified (Figure S2b) and will not be discussed in detail here.

The PCoA (Figures 4(a) and 4(b)) shows that the bacterial and fungal communities in the YC1, YC2, and YC3 groups can be well separated through the principal coordinate components, indicating that the samples of the same planting year have great similarity. Through the sample clustering tree (Figures 4(c) and 4(d)), YC3 and YC5 had higher similarity in the fungal community composition, whereas the results of bacteria were different with fungi based on the Bray-Curtis method. The samples of the YC1 group and the YC5 group were clustered into one class first, indicating that the changes in bacteria are fluctuating and may not be related to the continuous cropping years.

**Figure 7:**
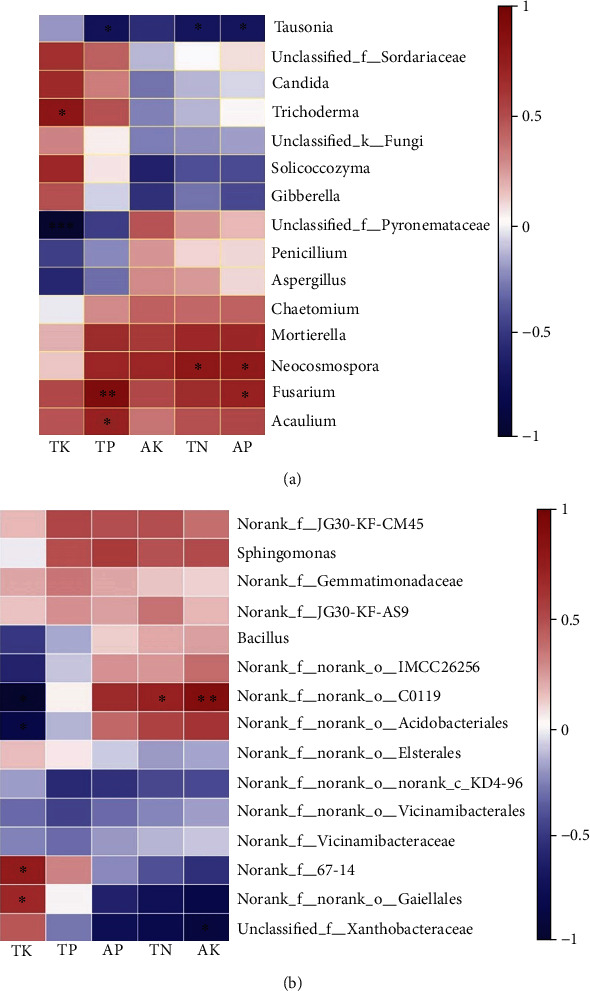
Correlation heat map of microbial classification and environmental factors. (a) Correlation heat map of the top fifteen genera (fungi) and soil nutrient content. (b) Correlation heat map of the top fifteen genera (bacteria) and soil nutrient content. The *x*-axis and the *y*-axis are environmental factors and species, respectively. The *R* values are shown in different colours in the figure. The right legend is the colour range of different *R* values. Significant values are shown as ^∗^0.01 < *P* ≤ 0.05, ^∗∗^0.001 < *P* ≤ 0.01, and ^∗∗∗^*P* ≤ 0.001.

Investigations of the microbial community in (rhizosphere) soil could provide new opportunities to explore the potential of antagonistic microorganisms in the suppression of plant pathogens [41]. At present, more studies have focused on detecting the roles of soil microbial communities and their relationships with soil environmental factors [42]. RDA analysis shows that the main environmental variables have a significant influence on the fungal community at the phylum level; in particular, the influence becomes greater as the continuous cropping years increase (Figure 6). The correlation between environmental factors and microbial communities was discussed by correlation heat map analysis at the genus level (Figure 7), and it indicated that *Fusarium* with significant pathogenicity was positively correlated with AP and TP. Farmers in China applied large amounts of commercial nitrogen and phosphoric fertilizers to achieve higher yields; however, excessive N and P fertilizers may be a disadvantage for plant growth [51, 52]. At the same time, *Trichoderma* which can control *Fusarium wilt* to a certain extent was positively correlated with TK [53]. We have concluded that fertilizer has an important influence on the regulation of key microorganisms during crop growth. Reasonable fertilization may help inhibit harmful pathogens, which is beneficial to control the continuous cropping obstacles of Chinese chives, especially Fusarium wilt.

## 5. Conclusion

This study represents the first use of high-throughput sequencing technology to analyse changes of microbial community structure in the soil during continuous cropping of Chinese chives. Sequencing of the fungal ITS region and the bacterial 16S rRNA region provided detailed insight into the microbial community patterns that develop dynamically in Chinese chive soil during continuous cropping. In this study, it indicated that short-term continuous cropping had no significant effect on the level of bacterial and fungal alpha-diversity in the Chinese chive field, but the Venn chart indicated that the proportion of shared bacterial OTUs in the three groups was significantly higher than that of fungi.

By comparing the bacterial and fungal differences at the phylum and genus levels, it indicated that some pathogenic fungi such as *Fusarium* appeared to have a significant increase, and the beneficial bacteria such as *Pseudomonas* decreased significantly, although it did not cause significant continuous cropping disorders. In the future, further research on soil microbes in Chinese chives should be conducted to explore the influence of different types and contents of chemical fertilizers on the microbial flora and disease occurrence. The interaction between the rhizosphere and microorganism in the soil environment should deserve more attention. It can provide further scientific guidance for the prevention and control of continuous cropping obstacles.

## Figures and Tables

**Table 1:** Soil nutrient content in soil under different continuous cropping years.

Groups	YC1	YC3	YC5
TN (%)	0.132 ± 0.001b	0.124 ± 0.002c	0.135 ± 0.00a
TP (%)	0.12 ± 0.001b	0.12 ± 0.00b	0.20 ± 0.004a
AP (mg/kg)	55.1 ± 0.08b	33 ± 0.08c	68.1 ± 0.05a
AK (mg/kg)	117 ± 0.16b	92.76 ± 0.03c	118.1 ± 0.05a
TK (g/kg)	16.1 ± 0.02b	17.1 ± 0.05a	17.1 ± 0.04a
PH	8.05 ± 0.04a	8.06 ± 0.05a	8.03 ± 0.07a
SOM (g/kg)	17.95 ± 0.11a	18.04 ± 0.1a	17.96 ± 0.32a

Values are means ± standard deviation (*n* = 3). Means followed by the same letter for a given factor are not significantly different (*P* < 0.01) according to one-way analysis of variance (ANOVA).

**Table 2:** Alpha-diversity index table.

Fungi	YC1	YC3	YC5
Sobs	430.67 ± 58.43a	497.67 ± 17.56a	495.0 ± 44.17a
Shannon	3.96 ± 0.140a	4.17 ± 0.089a	4.08 ± 0.09a
Simpson	0.045 ± 0.006a	0.037 ± 0.008a	0.036 ± 0.004a
Ace	453.77 ± 58.444a	524.78 ± 20.61a	532.52 ± 63.02a
Chao	455.47 ± 59.65a	532.63 ± 16.88a	537.12 ± 65.51a
Coverage	0.9995 ± 0.0002a	0.9988 ± 0.0002a	0.999 ± 0.0005a
Bacteria	YC1	YC3	YC5
Sobs	1954 ± 5.29a	1859 ± 69.18a	1908.7 ± 90.12a
Shannon	6.34 ± 0.05a	6.345 ± 0.117a	6.33 ± 0.157a
Simpson	0.0040 ± 0.0003a	0.0047 ± 0.0017a	0.0046 ± 0.0011a
Ace	2157.8 ± 18.65a	2078.2 ± 57.03a	2155.7 ± 60.61a
Chao	2181.7 ± 19.03a	2116.2 ± 81.98a	2166.3 ± 67.75a
Coverage	0.989 ± 0.0007a	0.989 ± 0.00007a	0.988 ± 0.0008a

Values are means ± standard error (*n* = 3). Means followed by the same letter for a given factor are not significantly different (*P* < 0.05) according to the Wilcoxon rank sum test. The number of OTUs, richness estimator Chao and ACE, and diversity estimator Shannon and Simpson were calculated at 3% distance.

## Data Availability

The (raw sequence reads) data used to support the findings of this study have been deposited in the (NCBI) repository (accession: PRJNA561880). This is an open access article distributed under the Creative Commons Attribution License, which permits unrestricted use, distribution, and reproduction in any medium, provided the original work is properly cited.

## Supplementary Materials

Supplementary Materials Table S1: the top 15 genus abundance table with significant differences. Figure S1: difference analysis of the phylum level by means of one-way ANOVA: (a) fungi; (b) bacteria (abundance > 1%). Figure S2: the community heat maps of (a) fungi and (b) bacteria at genus levels. The *x*-axis is the group name, the *y*-axis is the species name, and the abundance value of each group is the average of three replicates, which is showed by the colour block gradient changes of different species in the sample, and the right side of the figure is the value represented by the colour gradient.
