# Supplementary material for: Study on the Diversity of Fungal and Bacterial Communities in Continuous Cropping Fields of Chinese Chives (Allium tuberosum)
Source: Biomed Res Int. 2020 Dec 17;2020:3589758. doi: 10.1155/2020/3589758 (PMC7762660; doi:10.1155/2020/3589758)
Supplement: Supplementary Materials — Table S1: the top 15 genus abundance table with significant differences. Figure S1: difference analysis of the phylum level by means of one-way ANOVA: (a) fungi; (b) bacteria (abundance > 1%). Figure S2: the community heat maps of (a) fungi and (b) bacteria at genus levels. The x-axis is the group name, the y-axis is the species name, and the abundance value of each group is the average of three replicates, which is showed by the colour block gradient changes of different species in the sample, and the right side of the figure is the value represented by the colour gradient. [file 3589758.f1.docx]

## Supplementary Materials

Table S1：The top 15 genus abundance table with significant differences.

| **Fungi(genus)** | **YC1** | **YC3** | **YC5** | **P value** |
| --- | --- | --- | --- | --- |
| Tausonia | 9.792±1.63 | 12.82±7.498 | 2.691±0.771 | 0.01509 |
| Fusarium | 4.106±1.338 | 4.102±1.152 | 7.82±0.975 | 0.02711 |
| Unclassified_f__Pyronemataceae | 12.48±2.677 | 0.7852±1.117 | 0.3093±0.378 | 0.01375 |
| Neocosmospora | 2.92±0.527 | 1.299±0.475 | 4.785±2.536 | 0.04707 |
| Unclassified_f__Sordariaceae | 1.344±0.113 | 2.287±0.297 | 2.506±1.301 | 0.03844 |
| Unclassified_f__Microascaceae | 0.4325±0.182 | 0.6704±0.175 | 2.75±0.926 | 0.0465 |
| Thielavia | 1.587±0.358 | 0.3345±0.040 | 1.17±0.204 | 0.01113 |
| Humicola | 1.994±0.352 | 0.05388±0.0158 | 0.6284±0.334 | 0.01017 |
| Colletotrichum | 1.139±0.955 | 1.006±0.145 | 0.1854±0.0578 | 0.008026 |
| Eleutherascus | 1.694±0.402 | 0.2582±0.159 | 0.06018±0.0294 | 0.02052 |
| Microascus | 0.1211±0.0479 | 0.9146±1.148 | 0.7257±0.129 | 0.01421 |
| Exophiala | 0.09307±0.0280 | 1.255±0.139 | 0.09167±0.0179 | 0.001247 |
| Unclassified_p__Chytridiomycota | 0.5696±0.596 | 0.226±0.0789 | 0.5192±0.0809 | 0.04261 |
| Sagenomella | 0.6662±0.166 | 0.1407±0.0476 | 0.3058±0.217 | 0.03836 |
| Gibellulopsis | 0.4101±0.457 | 0.06018±0.0149 | 0.529±0.0536 | 0.002749 |
| **Bacterial** | | | | |
| Norank_f__norank_o__C0119 | 4.35±0.072 | 9.016±1.801 | 6.933±2.157 | 0.05908 |
| Norank_f__67-14 | 0.7933±0.146 | 1.663±0.239 | 1.608±0.5644 | 0.0238 |
| Norank_f__norank_o__Subgroup_2 | 1.832±0.413 | 0.569±0.258 | 1.105±0.2481 | 0.03508 |
| Nitrolancea | 0.7133±0.185 | 0.736±0.150 | 1.194±0.08243 | 0.02168 |
| Chujaibacter | 1.068±0.276 | 1.1±1.116 | 0.2507±0.125 | 0.04668 |
| Norank_f__LWQ8 | 0.2951±0.0872 | 1.12±0.303 | 0.6787±0.2359 | 0.04032 |
| Norank_f__norank_o__norank_c__AD3 | 0.4507±0.210 | 1.058±0.168 | 0.1989±0.095 | 0.007887 |
| Norank_f__norank_o__norank_c__bacteriap25 | 0.3458±0.1089 | 0.7663±0.140 | 0.3394±0.035 | 0.04074 |
| Solirubrobacter | 0.2788±0.006 | 0.442±0.0384 | 0.3696±0.105 | 0.02033 |
| Pseudomonas | 0.6236±0.221 | 0.3761±0.139 | 0.0562±0.0146 | 0.03682 |
| Norank_f__WWH38 | 0.1978±0.050 | 0.3404±0.0944 | 0.492±0.073 | 0.01685 |
| Unclassified_o__Solirubrobacterales | 0.3804±0.092 | 0.2064±0.0412 | 0.401±0.064 | 0.03232 |
| Unclassified_o__Saccharimonadales | 0.2929±0.016 | 0.2594±0.0181 | 0.399±0.036 | 0.01566 |
| Unclassified_f__Comamonadaceae | 0.3069±0.131 | 0.3729±0.0245 | 0.1513±0.041 | 0.008166 |
| Unclassified_c__Ktedonobacteria | 0.03891±0.003 | 0.01513±0.005 | 0.5361±0.2663 | 0.009651 |

p represent phylum, c class, o order, f family. Significant differences in the different groups were evaluated with one-way ANOVA, and P values were corrected for multiple comparisons using the false discovery rate (FDR).


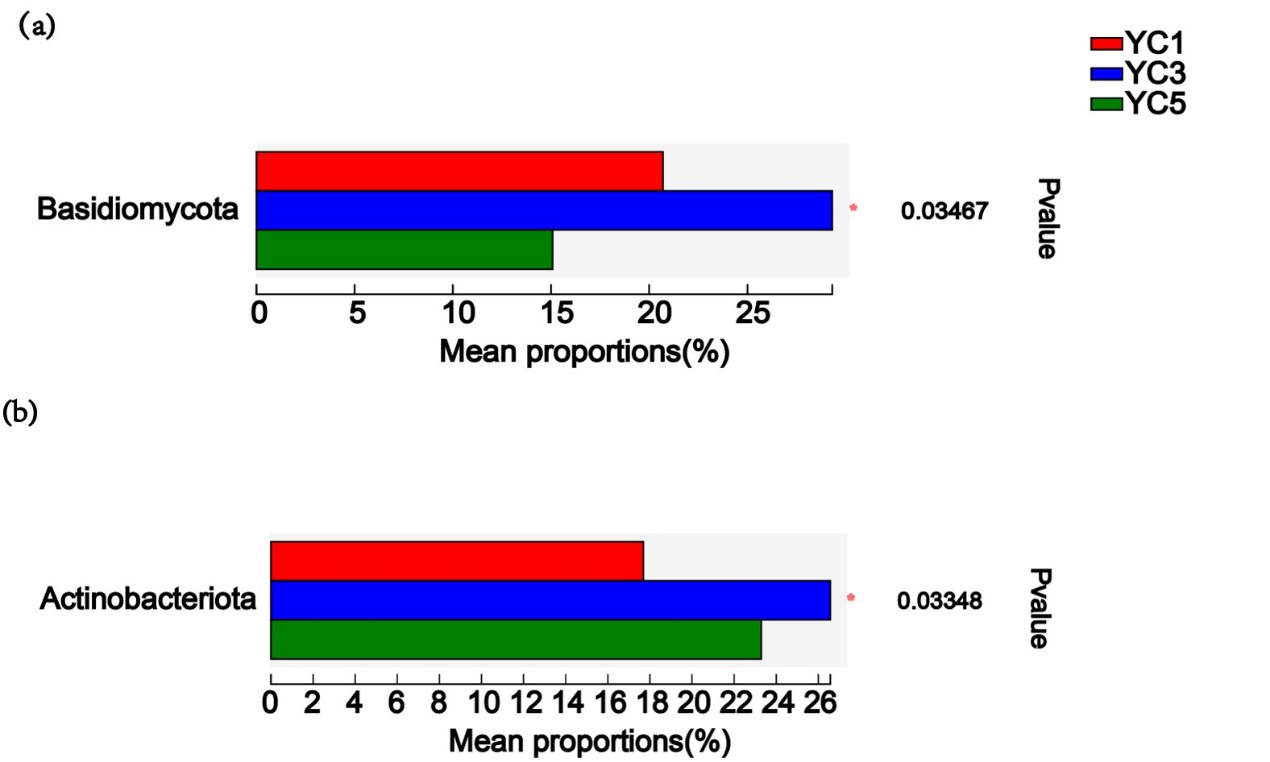


Fig S1：Difference analysis of the phylum level by means of one-way ANOVA.(a) fungi; (b) bacteria (abundance> 1%).


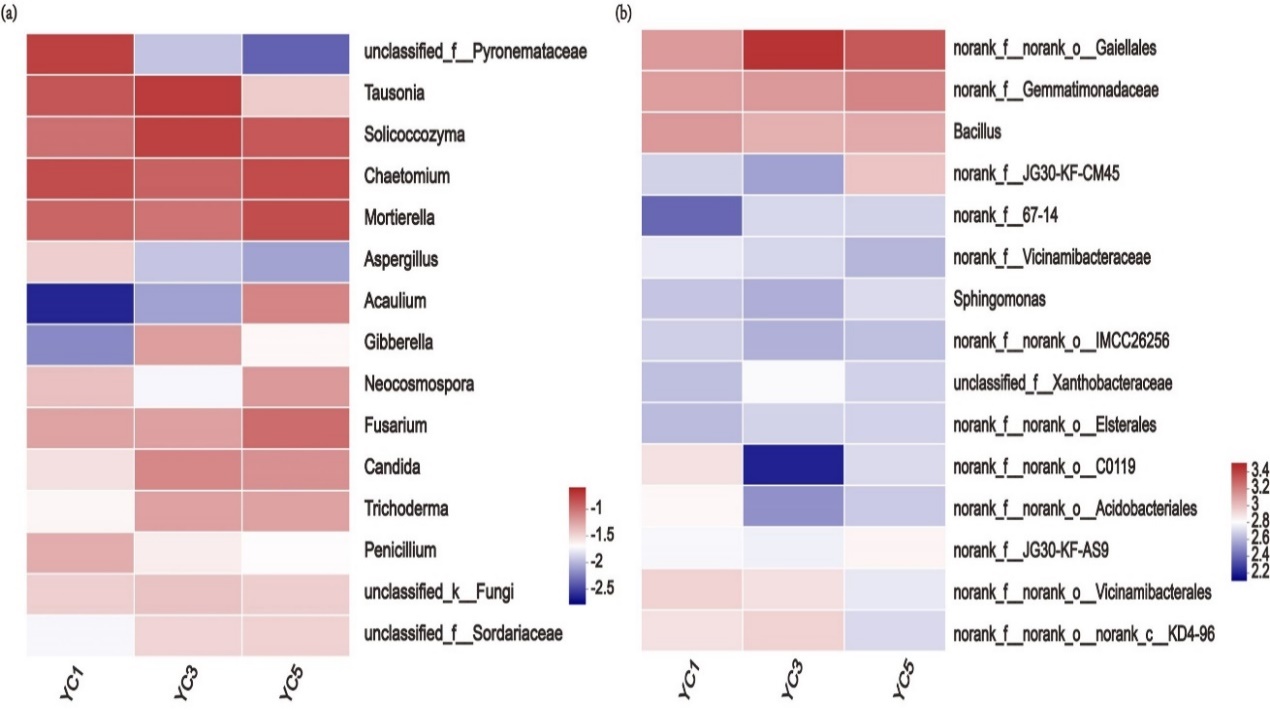


Fig S2: The community heat maps of fungi (a) and bacteria (b) at genus levels. The X-axis is the group name, the Y-axis is the species name, and the abundance value of each group is the average of three replicates, which is showed by the colour block gradient changes of different species in the sample, and the right side of the figure is the value represented by the colour gradient.
